# Electroacupuncture of the trigeminal nerve causes *N*-methyl-D-aspartate receptors to mediate blood-brain barrier opening and induces neuronal excitatory changes

**DOI:** 10.3389/fncel.2022.1020644

**Published:** 2022-10-13

**Authors:** Peng Gong, Shanshan Zhang, Li Ren, Jiangsong Zhang, Yibin Zhao, Xuqing Mao, Lin Gan, Hao Wang, Congcong Ma, Yubo Lin, Qinyu Ye, Kecheng Qian, Xianming Lin

**Affiliations:** Key Laboratory of Acupuncture and Neurology of Zhejiang Province, Department of Neurobiology and Acupuncture Research, The Third Clinical Medical College, Zhejiang Chinese Medical University, Hangzhou, China

**Keywords:** blood-brain barrier, electroacupuncture, trigeminal nerve, NMDA receptor, MK-801, electrophysiology

## Abstract

The blood-brain barrier (BBB) is an important structure for maintaining environmental stability in the central nervous system (CNS). Our previous study showed that specific parameters of electroacupuncture (EA) at the head points Shuigou (GV26) and Baihui (GV20) can open the BBB; however, the mechanism by which stimulation of body surface acupuncture points on the head results in peripheral stimulation and affects the status of the central BBB and the neuronal excitatory changes has not been elucidated. We used laser spectroscopy, the *In Vivo* Imaging System (IVIS), immunofluorescence and immunoblotting to verified the role of the trigeminal nerve in BBB opening during EA, and we applied the central *N*-methyl-D-aspartate (NMDA) receptors blocker MK-801 to verify the mediating role of NMDA receptors in EA-induced BBB opening. Next, electroencephalogram (EEG) and *in vivo* calcium imaging techniques were applied to verify the possible electrical patterns of BBB opening promoted by different intensities of EA stimulation. The results showed that the trigeminal nerve plays an important role in the alteration of BBB permeability promoted by EA stimulation of the head acupoints. Brain NMDA receptors play a mediating role in promoting BBB permeability during EA of the trigeminal nerve, which may affect the expression of the TJ protein occludin, and thus alter BBB permeability. The analysis of the electrical mechanism showed that there was no significant change in the rhythm of local field potentials (LFP) in different brain regions across frequency bands immediately after EA of the trigeminal nerve at different intensities. However, the local primary somatosensory (S1BF) area corresponding to the trigeminal nerve showed a transient reduction in the delta rhythm of LFP with no change in the high-frequency band, and the action potential (spike) with short inter spike interval (ISI) varied with EA intensity. Meanwhile, EA of the trigeminal nerve resulted in rhythmic changes in calcium waves in the S1BF region, which were influenced by different EA intensities. This study provides a research perspective and a technical approach to further explore the mechanism of EA-induced BBB opening and its potential clinical applications.

## Introduction

The blood-brain barrier (BBB) is an important structure that maintains the stability of the internal environment of the central nervous system (CNS) (Daneman and Prat, 2015). The BBB is mainly composed of brain capillary endothelial cells (EC), tight junction proteins (TJ), pericytes (PC) (Hall et al., 2014), basement membranes (BL), and astrocyte (AS) endfeet (Girouard et al., 2010) among others (Zhao et al., 2015; Winkler et al., 2021). Because of its structural and functional characteristics, BBB “shuts down” several drugs used to treat neurological diseases (Zlokovic, 2008; Abbott and Friedman, 2012), such as exogenous nerve growth factor (NGF), which becomes a major obstacle during treatment (Miller, 2010; Mahringer et al., 2011). According to statistics, more than 90% of neurotherapy drugs lose their effectiveness because of the difficulty in getting into the brain. Therefore, the BBB is a “double-edged sword.”

Currently, several approaches for promoting the BBB opening are being explored (Li et al., 2021). When the BBB acts as a hurdle to drug delivery, increasing BBB penetration is the key. Current strategies for the transient disruption of BBB TJs include the application of osmotic agents, angiogenic molecules such as vascular endothelial growth factor (VEGF-A) (Lundy et al., 2019), chemical agents such as autoantibodies (GRP78), physical methods such as magnetic resonance-guided focused ultrasound (MR-FUS) (Abrahao et al., 2019; Chen et al., 2019), and exocytosis of pump *P*-glycoprotein (gp) and breast cancer resistance protein (BCRP1) inhibitors (Tournier et al., 2015). BBB can also be used as a Carrier for Drug Delivery: Harnessing Endogenous Transcytosis Systems, including exosome-based delivery systems (Morad et al., 2019), nanoparticle-based delivery systems (Anraku et al., 2017; Lam et al., 2018), and virus-based delivery systems (Deverman et al., 2018). However, some of these methods to open the BBB are still in the experimental stage, and others are challenging, such as the toxicity associated with nanoparticle delivery systems, which hinders their further application (Aragon et al., 2017). Currently, several other methods are also difficult to be applied widely in clinical settings. Therefore, the exploration of effective, reversible, and non-invasive methods to enhance BBB permeability to promote drug delivery to the brain has attracted clinical attention. Electroacupuncture (EA) is a modern acupuncture modality that involves the application of pulsating electric currents to acupuncture needles for stimulation of acupoints. Previous studies have found that specific parameters of electroacupuncture (EA) of Shuigou (GV26) and Baihui (GV20) points can open the BBB instantly, reversibly, and without side effects (Zhang J. S. et al., 2018; Zhang et al., 2020). EA has been widely used in basic acupuncture research and clinical treatment because the acupoints can be selected according to the disease and the stimulation parameters can be easily and clearly controlled (Fang et al., 2017).

We found that EA of GV26 + GV20 opens the BBB, but non-head acupoints do not play a significant role, and the mechanisms by which head and body acupuncture stimulation results in peripheral stimulation and affects the central BBB status have not yet been elucidated. As GV26 and GV20 are located in the sensory region of the trigeminal nerve, we suggest that the trigeminal nerve may play an important role in EA stimulation of head acupoints. Furthermore, some studies have shown that central NMDA receptors mediate BBB opening through peripheral stimulation and influence BBB disruption (Beard et al., 2011; Neuhaus et al., 2011; Hogan-Cann et al., 2019), we also have conducted the corresponding experimental design verification and preliminary exploration of the association mechanism level. Preliminary research have screened for different frequencies and durations of EA at an early stage (Zhang et al., 2020), however, the electrophysiological mechanisms behind the large differences in BBB opening with and without EA and the different intensities of EA also need to be elucidated.

Therefore, this experiment was divided into two major parts: the first part involved the identification of the role of trigeminal nerve in promoting BBB opening during EA of head acupoints, and the change in the permeability of BBB with EA, which was observed by excision of the infraorbital nerve (the largest branch of the trigeminal nerve) and trigeminal ganglion block. The mechanism of BBB opening by EA was initially explored by comparing the changes in the brain *N*-methyl-D-aspartate (NMDA) receptor NR1 and brain endothelial cell TJ protein occludin in each group, and then the NMDA receptor antagonist MK-801 was applied to verify whether MK-801 could reverse NMDA receptor action (Andras et al., 2007; Chen et al., 2016). The second part involved the application of electroencephalogram (EEG) (Bian et al., 2018) and *in vivo* calcium imaging techniques (Li et al., 2017), following the stimulation of the acupoints that were screened in the first part, on the premise that different intensities of EA can promote different degrees of neuronal activity, leading to different degrees of BBB opening. By observing the rhythmic oscillations of neurons generated by EA and the fluctuations in calcium ion flow in individual neurons, we found that EA promotes open electrical regulatory patterns of the BBB under specific parameters.

## Materials and methods

### Experimental animals

All procedures in this study were performed in accordance with the National Institutes of Health Guide for the Care and Use of Laboratory Animals. The experimental protocol was approved by the Animal Health Care Use Committee of Zhejiang University of Traditional Chinese Medicine and complied with internationally recognized ethical standards. All effort were made to reduce animal suffering, minimize the number of animals used, and utilize alternatives to *in vivo* techniques whenever possible. The first part of the experiment, which involved screening of different acupuncture points along with the initial mechanistic exploration and EEG were carried out on 12-week-old male Sprague Dawley rats; however, for the *in vivo* calcium ion imaging detection, the rats failed to express GCaMPs effectively due to viral transfection and C57 mice were therefore subsequently used for the experiment. The animals were kept at 25 ± 2°C and 50 ± 10% humidity under a 12 h light/dark cycle. Food and water were provided by the Experimental Animal Center of Zhejiang University of Traditional Chinese Medicine, Hangzhou, Zhejiang Province, China.

### Electroacupuncture stimulation

The effects of EA stimulation at different acupoints on BBB permeability were investigated. Rats were randomly divided into the control, EA ZuSanli (ST36), and EA GV26 + GV20 groups with the following parameters: 2/100 Hz, 3 mA, 6/6 s, 40 min (*n* = 12 rats per group), and 2% EB in saline (E2129-10G, Sigma-Aldrich, Shanghai, China) was injected into rats through the tail vein using an indwelling needle (2 ml/kg). In the EA group, a needle (Beijing Zhongyan Taihe Medical Equipment Co., Ltd., Beijing, China) measuring 13 mm long and 0.13 mm in diameter was inserted at GV20 and a needle 7 mm long and 0.07 mm in diameter was inserted at GV26. The needle was then stimulated for 40 min with an acupuncture point nerve stimulator (HANS-200; Jinsheng, Nanjing, China) at an intensity of 3 mA. The control group did not receive EA treatment and received only a 2% Evans blue (EB) saline injection through the tail vein. All the tests were conducted from 6:30 am to 8:30 pm GMT. The EB content in the brain tissue of 6 rats in each group was measured separately, and the distribution of EB and c-Fos in the 6 rats was observed using the *In Vivo* Imaging System (IVIS) and fluorescence microscopy.

To clarify the effect of trigeminal nerve in EA stimulation for BBB opening, rats were divided into control, EX-HN6 (located in the non-trigeminal distribution area of the head), GV26 + GV20 (located in the trigeminal distribution area of the head), and GV26 + infraorbital nerve stimulation point (the largest branch of the trigeminal trunk) groups with the same parameters as aforementioned (*n* = 10 rats per group). EB content was measured in six rats per group, and four rats were observed using fluorescence microscopy for the distribution of EB and c-Fos. To verify the role of the trigeminal nerve in EA-induced BBB opening, rats were randomly divided into the GV26 + infraorbital nerve stimulation point group, infraorbital nerve resection group, and trigeminal ganglion block group (*n* = 10 rats per group). EB content was measured in six rats in each group, and the distribution of EB with c-Fos was observed in 4 rats using fluorescence microscopy.

This study aimed to clarify the role and mechanism of NMDA receptors in trigeminal nerve-mediated EA opening of the BBB. To observe the effect of trigeminal EA stimulation on NR1 and TJ protein occludin, rats were randomly divided into control, EX-HN6, GV26 + GV20, and GV26 + infraorbital nerve stimulation point groups with the same parameters as mentioned before (*n* = 12 rats per group). Six rats from each group were observed for fluorescence intensity and structural changes of NR1 and occludin after EA. Six rats were immunoblotted to determine the effects of EA on NR1 and occludin protein expression. To reverse verify whether brain NMDA receptors play a role in trigeminal nerve-mediated EA opening of the BBB, rats were randomly divided into control, GV26 + infraorbital nerve stimulation point, and GV26 + infraorbital nerve stimulation point + NMDAR antagonist MK-801 (M107-5MG, Sigma-Aldrich, Shanghai, China) groups with the aforementioned parameters (*n* = 16 rats per group). Rats were injected intraperitoneally with MK-801 (0.2 mg/kg) and then waited for 10 min before EA stimulation. The EB content was measured in 6 rats in each group, and the distribution of EB and c-Fos, as well as the fluorescence intensity and structural changes in NR1 and occludin were observed in four rats using immunofluorescence.

To investigate the mechanism of neuronal excitability alterations in the degree of BBB opening after EA stimulation of the trigeminal nerve with different current intensities, the rats were randomly divided into control, anesthesia, EA 1 mA, EA 3 mA, and EA 5 mA groups with the same parameters as aforementioned, anesthesia group were deeply anesthetized with sodium pentobarbital (50 mg/kg) (*n* = 16 rats per group). The effects of EA on NR1 and occludin protein expression were also investigated. To study the possible patterns of electrical modulation characteristics of trigeminal nerve stimulation by EA with different current intensities, we performed EEG, and the rats were randomly divided into a self-made electrode (similar to the ECoG) group, in which the electrode was implanted in the prefrontal, central, and occipital regions, and an intracranial electrode group, in which the electrode was implanted in the primary somatosensory (S1BF) area of the sensory cortex (EA stimulation parameters: 100 Hz, 6/6 s, 10 min; fixed stimulation points: GV26 + infraorbital nerve stimulation point, *n* = 5 rats in each group). Electroencephalography (EEG), local field potentials (LFP), and spike tests were performed before and after EA. Finally, we measured the calcium ion flow in S1BF, the corresponding brain region of glutamatergic neurons, to detect calcium ion flow in the trigeminal nerve. The AAV2/9-CaMKIIa-GCaMP6f-WPRE-pA virus (Taitool Bioscience, Shanghai, China) as injected into the S1BF area of the sensory cortex of C57 mice. We then performed EA stimulation in the 1, 3, and 5 mA groups with the abovementioned parameters (*n* = 4 mice per group) to observe the possible alterations in the excitability of glutamatergic neurons in layer 2/3 of the S1 cortex after EA of the trigeminal nerve.

### Brain perfusion

After EA, the rats were deeply anesthetized with sodium pentobarbital (50 mg/kg). The animals were then perfused with 0.9% saline through the left ventricle until the colorless fluid was obtained from the right atrium. After decapitation, the brain was removed, placed in liquid nitrogen for rapid freezing, moved to –80°C, and stored. For immunofluorescence and immunohistochemistry, rats were then perfused with 4% PFA. After the rat’s body and limbs were stiffened, the animal was decapitated, the brain was removed, placed in paraformaldehyde, and fixed for 24 h.

### Blood-brain barrier permeability detections

First, EB content was measured using a multifunctional enzyme standard analyzer (SpectraMax M5, Molecular Devices Co., Radnor, PA, United States). Cerebral cortex (stripped of the white matter) were taken from rats brain, weighed, and placed in 50% trichloroacetic acid solution, homogenized, centrifuged (10,000 r/m, 20 min), and the supernatant at a ratio of 1: 3 ethanol. The OD values were measured by a multifunctional enzyme analyzer (excitation wavelength 620 nm, emission wavelength 680 nm). The EB content (ng/g) in brain tissue was derived from the extrinsic standard curve of EB solution (0–50 ng/ml). The expression of EB on the brain surface was then observed by IVIS, and the samples were placed in a small animal physiological signal telemetry device to measure the intensity of EB fluorescence in the Cy5.5 channel by IVIS. To observe EB by laser confocal microscopy, frozen 30 μm thick brain slices with 3 mm between each slice were selected. Photographs were taken with a laser confocal microscope (LCM, Nikon Eclipse Ti, Japan) in the Cy5 channel. EB expression was observed with a digital pathology section (fluorescence) scan analyzer.

### Infraorbital nerve excision

Rats were fed regularly and rationed with water, and the experiment started a week after feeding. After respiratory anesthesia with 4% isoflurane, and was maintained with 1–2% isoflurane, the rats were placed in a lateral position with the head and limbs fixed. 3% iodophor was used to disinfect the rat, and a sterile cavity was placed on the cheek of the rat at the surgical site. An incision of approximately 0.5–0.7 cm in length was made along the anterior 1/3 of the lower edge of the cheekbone of the rat (near the dorsal surface of the nose), approximately 45° below the sagittal front of the medial canthus of the eye, in a straight line of about 1 cm. After rapid stripping of the zygomaticus muscle with forceps, the distal trunk of the infraorbital nerve, where the tactile sensory nerves converge into a bundle, can be found and excised with ophthalmic scissors. After surgery, the wound was rinsed with saline and the incision is layered with sutures. Intramuscular injection of penicillin 150,000μ/kg to prevent infection. Feeding for 7 days and the sensory function of the trigeminal nerve was observed. The sensation of bilateral whisker pads in each group of rats was detected by acupuncture needle. No response or sluggish response was considered to indicate successful modeling, and then EA stimulation points with specific parameters.

### Trigeminal ganglion block

The rats were respiratory anesthetized with 4% isoflurane. Anesthesia was maintained with 1–2% isoflurane in air, the rats were placed in a lateral recumbent position, sterilized with 3% iodine, and an incision of approximately 0.5 cm long was made along the anterior 1/3 segment of the inferior border of the cheekbone (near the dorsal surface of the nose). The perimuscular fascia was bluntly separated to expose the infraorbital foramen, and the infraorbital nerve was seen to fan out behind the infraorbital foramen. The purpose of the operation to expose the suborbital nerve stem and infraorbital foramen is to ensure the accuracy of the operation. Prepare 2% lidocaine (0.5 ml/kg) and inject it into the infraorbital foramen (around the infraorbital nerve) to ensure correct positioning. After making sure there was no drug leakage, rinse the wound with saline and sew the incision layer by layer. Since lidocaine has a time window effect, subsequent EA point stimulation with specific parameters should be performed immediately.

### Immunofluorescence staining

Brain tissue was taken, 4% paraformaldehyde was placed for 24 h and then 15%, 30% sucrose step dehydration. The brain was coronal or sagittal sectioned at 30 μm thickness on a Leica CM1950 cryostat. Occludin protein thermally repaired with citrate solution, and slices were closed with 0.3% Triton X-100 and 5% goat serum in 0.01 M phosphate-buffered saline (PBS) for 1 h, then incubated overnight at 4°C with the following concentrations of primary antibody 12–16 h: rabbit anti-c-Fos (1:200, Cell Signaling, the 2250S), rabbit anti-occludin (1:300, Invitrogen, 71-1500), rabbit anti-NR1 (1:600, Abcam, ab17345), diluted in 5% goat serum and 0.3% Triton X-100 in PBS. After rinsing, goat anti-rabbit fluorescent secondary antibody (1:400; 488 nm, Jackson, 111-545-144) was diluted in PBS containing 5% goat serum and 0.3% Triton X-100 in a constant temperature water bath at 37°C for 1 h. After secondary antibody incubation, brain slices were washed, sealed with Fluoroshield Mounting Medium with DAPI, and observed with a Zeiss Axio Imager M2 and Nikon Eclipse Ti. c-Fos protein were quantified using Image-J and photoshop software.

### Western blot analysis

After EA, the cerebral cortex of each group of rats were taken, total protein of samples was extracted, protein content was determined by BCA method, protein pre-denaturation, electrophoresis, gel cutting and membrane transfer were performed. 0.5% defatted milk powder was used as the closure solution, rabbit anti-β-actin (1:2500 Cell signal, 5125S) and rabbit anti-occludin (1:1000, Invitrogen, 71-1500), rabbit anti-NR1 (1:600 Abcam, ab17345) overnight at 4°C and rewarmed at 37°C, The membrane was incubated with an HRP-conjugated goat anti-rabbit/mouse (H + L) secondary antibody (1:5000 Bioker Biotechnology) diluted 1:5000 in TBST for 2 h at room temperature. Wash the membranes and add the chromogenic agent to use. Finally, protein bands were observed using the ImageQuant LAS 4000 system and semi-quantified using Image-J software.

### Electroencephalography

#### Self-made electrode implantation

The experiment was started after one week of acclimatization feeding. After anesthesia with ethyl carbamate (1.2 g/kg i.p.), the rats were fixed on the stereotaxic apparatus, their eyes glued to erythromycin ointment to prevent blinding by bright light, and placed on operating tables at 37°C to maintain their body temperature. The cranial nail was implanted with a dental drill in the prefrontal (BL: +2.0 mm ML: 2.25 mm), central (BL: –3.0 mm ML: 2.75 mm), and occipital regions (BL: –7.0 mm ML: 2.75 mm), and the cranial nail was implanted as a ground wire with the polar wire wrapped around the nail prepared in advance (Self-made electrode implantation DV: 0.8–1.2 mm). After implantation, homemade electrodes were fixed with dentin cement and EEG signals were detected on the spot.

#### Intracranial electrode implantation

The preliminary work is the same as homemade electrode implantation, except that the intracranial electrode is implanted in the corresponding observation brain area, and the main sensory cortex area (S1 layer II/III) is selected and positioned as BL: –3.0 mm ML: 5.5 mm DV: 1.0 mm. The 4 × 2 array of micropolar wires (made of nickel-chromium, 35 μm in diameter, 200 μm distance between recording lines) was slowly implanted and secured with surface cranial nails and dental cement.

### *In vivo* calcium imaging

C57BL/6 mice (6–8 weeks) were first anesthetized with sodium pentobarbital (0.03 ml/g, intraperitoneally injected with 3% solution), the head was fixed in a stereotaxic frame, and the mice were pretreated with dexamethasone (0.2 mg/kg, s.c.) and carprofen (5 mg/kg, s.c.) to prevent surgically induced pain, swelling and inflammation. Rinse and dry the skull with 3% hydrogen peroxide, continuously wipe the skin with iodine volts and 75% ethanol, and make an incision of about 1.5 cm on the scalp with a sterile scalpel without removing the scalp. The dura mater of the target area (about 1 mm^2^) was exposed and picked up by lifting the bone flap, attaching a glass pipette (tip size: 0.3–0.5 mm) to a 10-μl micro syringe, and pulling the glass pipette (Sutter Instrument, item number: BF120-94-10, model number: P87) with a micropipette puller (Sutter Instrument). Draw 0.6 μl of virus (Taitool, AAV2/9-CaMKIIa-GCaMP6f-WPRE-pA, potency dilution to 4 × 10^12^ V.G./ml) into the glass pipette by negative pressure, seal the pipette on the brain surface and inject into the virus at a rate of 60 nl/min. After injection, a 0.1 mm glass coverslip (Grobel, Shanghai, China) was placed on the cortex of the opened cranial window, a cranial cover was made with 1454 glue, and the glue is accelerated by a dental cement plasticizer and the scalp is stitched. The microscope was attached to the substrate until there was a gap of about 2 mm between the overlay and the microscope lens. After a clear vascular pattern is observed, the substrate is permanently immobilized with dental cement to cover the circumference of the cranial window. Calcium imaging was recorded with a head microscope (Inscopix; objects 2 mm in diameter; LED power: 0.6–1.0 mW) and images of 20 Hz were captured with the nVista acquisition software (Inscopix). The depth of imaging was selected by adjusting the focal length of the microscope until a clear astrocytic calcium signal was observed in the ΔF/F image. Principal and independent component analysis (PCA-ICA) was applied to the spatiotemporal data matrix of ΔF/F0 using Inscopix Data Processing software. Glutamatergic neuronal cells detected, their spatial location and corresponding calcium traces were extracted from the images and used for the analysis of experimental results. After the experiment, the transfection of virus injection can be observed by Confocal microscopy, and the transfection success rate can be calculated by image.

### Statistical analysis

The data in the graph are expressed as mean ± SEM. Statistical analyses were performed using IBM SPSS 20.0. One-way analysis of variance (ANOVA) and Tukey’s *post hoc* test were used to compare ≥ 3 groups. Two-tailed Student’s *t*-test was used to compare the two groups. The comparison results were considered significantly different when *P* was < 0.05.

## Results

### Electroacupuncture at the head acupoints had a more significant effect on promoting blood-brain barrier permeability than non-head points

**FIGURE 1:**
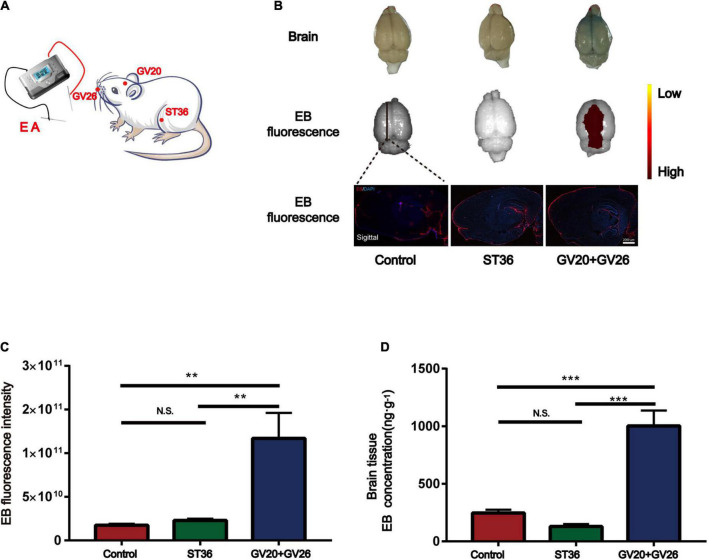
Electroacupuncture (EA) at the head acupuncture points had a more significant effect on promoting BBB opening than the non-head points. **(A)** Schematic diagram of EA at different points. **(B)** The first row shows the degree of brain EB penetration of each group under naked eye observation. Distribution of brain EB in each group was observed using IVIS (Color Scale: Min = 2.25 e^9^, Max = 2.96 e^11^). The third row shows the fluorescence intensity and distribution of EB in each group under the brain sagittal slice. **(C)** EB fluorescence intensity of cerebral cortex in each group under IVIS; **P < 0.01, GV20 + GV26 group vs. control group, ST36 group; *P* > 0.05, control group vs. ST36 group, *n* = 6 per group. **(D)** EB concentration in the cerebral cortex of each group; ***P < 0.001, GV20 + GV26 group vs. control group and ST36 group; *P* > 0.05, control group vs. ST36 group, *n* = 6/group.

We applied specific parameters of EA to stimulate the head and non-head acupuncture points to observe the BBB opening. Figure 1A shows the schematic diagram of different acupuncture points stimulated by specific parameters of EA. Figure 1B shows that EB fluorescence in the cortical layer of rats stimulated by EA was observed with the naked eye, and EB fluorescence in the sagittal section of the brain was similar to that observed with the naked eye. We found a significant difference in cerebral cortex EB fluorescence values (detected by IVIS) between the EA GV26 + GV20 group and the control and ST36 groups (*P* < 0.01) (Figure 1C). The EB content (ng/g) extracted from the cerebral cortex of the rats in each group was measured spectrophotometrically by enzymatic standardization, and a highly significant difference (*P* < 0.001) was observed in the cortical EB content of rats in the EA GV26 + GV20 group relative to the control and ST36 groups, as seen in Figure 1D. The differences in cortical EB content, as well as fluorograms, indicated that the BBB opening was more obvious with specific EA parameters of head acupuncture points compared to non-head acupuncture points.

### Electroacupuncture stimulation of the trigeminal nerve in the head region to promote blood-brain barrier opening had a more pronounced effect

**FIGURE 2:**
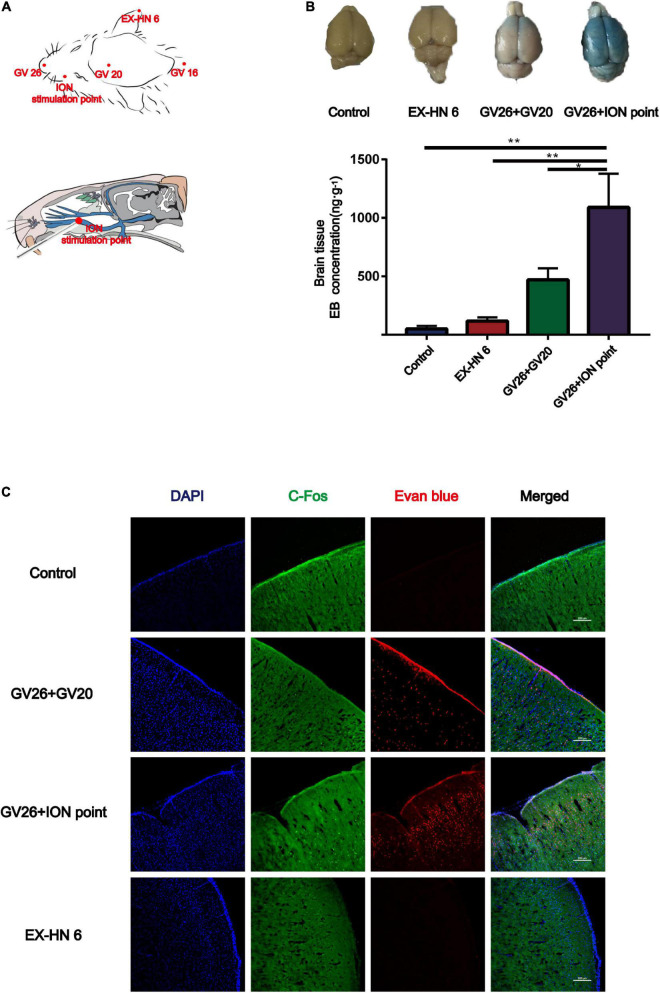
In the head region, EA stimulation of the trigeminal nerve promoted BBB opening more significantly. **(A)** Schematic diagram of the head acupoint and infraorbital nerve (ION) stimulation point (the largest branch of the trigeminal nerve, located in the upper 1/3 of the lower margin of the zygomatic bone of the rat right cheek, close to the back of the nose). **(B)** The upper part shows the degree of EB penetration in the brain of each group under naked eye observation, the cortex of each group; **P < 0.01, GV26 + ION point group vs. control group and EX-HN6 group; *P < 0.05, GV26 + ION point group vs. GV20 + GV26 group, *n* = 6/group. **(C)** EA of head different acupoints, cerebral cortex EB, and c-Fos fluorescence expression. The GV26 + ION point group had the most significant expression.

Since the GV20 and GV26 points are located in the trigeminal nerve distribution area, the role of trigeminal nerve in EA stimulation of the head points to open the BBB was considered. Figure 2A shows the location of the different head points and the infraorbital nerve (the largest branch of the trigeminal nerve). The first row of Figure 2B shows that rats were divided into the control, EX-HN6, GV26 + GV20, and GV26 + infraorbital nerve stimulation point groups and were treated with specific parameters (frequency 2/100 Hz, 3 mA intensity, interval 6/6 s) immediately after EA intervention. The second row shows that the EB content (ng/g) in the cerebral cortex of rats in each group was measured using enzyme standardization spectrophotometry, and the results were consistent with those observed with the naked eye. The GV26 + infraorbital nerve stimulation point group showed significantly different results from that of the control and EX-HN6 groups. Figure 2C shows cortical EB permeation through the BBB was significantly increased in the cortex of the rats in the GV26 + ION point group compared to the control and EX-HN6 groups. c-Fos was activated extensively in the GV26 + ION point group, and the expression of c-Fos was higher than that in the other groups. By observing the EB content through cortex and neuronal c-Fos activation after EA intervention at selected points in the trigeminal and non-trigeminal nerve distribution areas, it was found that EA of the trigeminal nerve had a more pronounced effect on BBB opening, and the neurons were widely activated.

### Validation of the role of trigeminal nerve in electroacupuncture-induced blood-brain barrier opening by infraorbital neurectomy and trigeminal ganglion block

**FIGURE 3:**
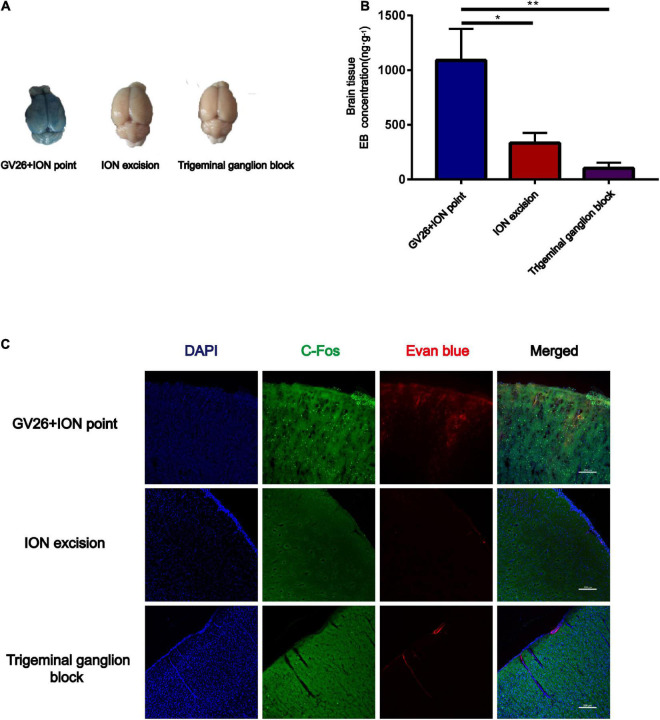
Verification of the role of trigeminal nerve in promoting BBB opening by infraorbital nerve excision and trigeminal ganglion block. **(A)** Shows the degree of brain EB penetration of each group under naked eye observation. **(B)** Shows EB concentration in the cerebral cortex of each group, *P < 0.05, GV26 + ION point group vs. ION excision group; **P < 0.01, GV26 + ION point group vs. trigeminal ganglion block group, *n* = 6/group. **(C)** The cerebral cortex EB and c-Fos fluorescence expression of the different groups. The ION excision group and trigeminal ganglion block group cerebral cortex EB and c-Fos fluorescence expression was significantly decreased.

To verify the role of trigeminal nerve in EA-stimulated BBB opening, we performed infraorbital neurectomy and trigeminal ganglion block in rats, followed by EA stimulation to observe BBB opening, found that the EB content (ng/g) extracted from the cerebral cortex of rats in each group with the same trend observed with the naked eye. The permeation of EB content through the BBB was significantly lower in the trigeminal ganglion block group compared to the GV26 + ION point group, with a highly significant difference (*P* < 0.01) (Figures 3A,B). Figure 3C indicates that after trigeminal ganglion block and infraorbital nerve excision with EA stimulation, EB permeation through BBB was significantly lower in the trigeminal ganglion block group and the trigeminal nerve excision group compared to the GV26 + ION point group. Further, the cortical neuronal c-Fos distribution was decreased and the activation level was significantly lower compared to the GV26 + infraorbital nerve stimulation point group. This suggests that a major part of the BBB opening induced by EA stimulation of the head acupoints may act through the conduction of the trigeminal nerve.

### Role of *N*-methyl-D-aspartate receptors in electroacupuncture stimulation of the trigeminal nerve to promote blood-brain barrier opening and the effect on tight junction occludin

**FIGURE 4:**
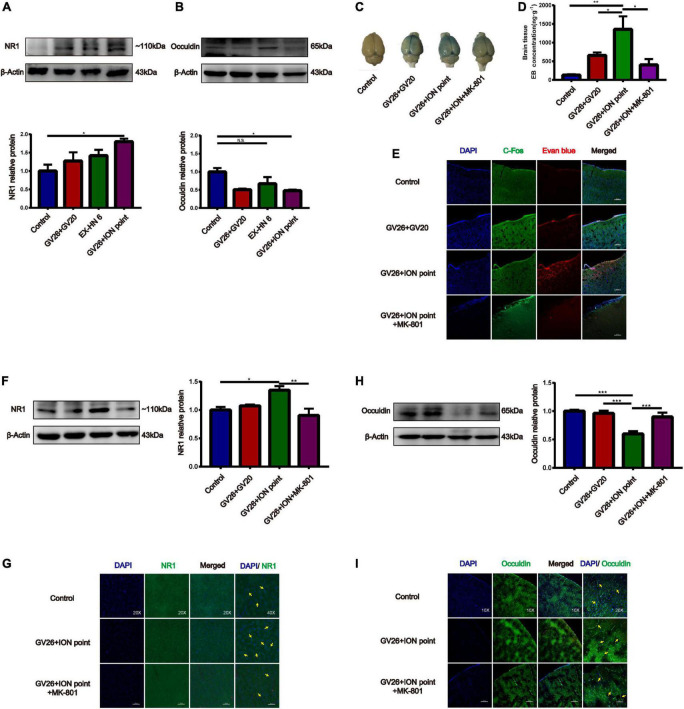
The role of NMDA receptors in EA stimulation of the trigeminal nerve to promote BBB opening and the effect on TJ occludin. **(A)** NR1 protein expression induced by EA at different head acupoints; Compared to the control group, NR1 protein expression was increased in all the EA groups, but the increase was most significant in the GV26 + ION group, *P < 0.05, GV26 + ION point group vs. control group, *n* = 6/group. **(B)** Occludin protein expression induced by EA at different head acupoints; occludin protein expression was decreased in the GV20 + GV26 group and GV26 + ION group, *P < 0.05, GV26 + ION point group vs. control group, *P* > 0.05, control group vs. EX-HN6 group, *n* = 6/group. **(C)** Shows the degree of brain EB penetration of each group under naked eye observation. **(D)** When NMDA receptor blocker MK-801 was used before EA of the trigeminal nerve, EB concentration in the cerebral cortex was decreased; *P < 0.05, GV26 + ION point group vs. GV26 + ION point + MK-801 group, *n* = 6/group. **(E)** The cerebral cortex EB and c-Fos fluorescence expression of the different groups, EA GV26 + ION group cerebral cortex EB and c-Fos. **(F)** EA GV26 + ION group NR1 protein expression was significantly decreased after using MK-801, **P < 0.01, GV26 + ION point group vs. GV26 + ION point + MK-801 group, *n* = 6/group. **(G)** EA GV26 + ION group cerebral cortex NR1 fluorescence expression(green, surrounding neuronal cytosol) was significantly decreased after treatment with MK-801 (yellow arrows). **(H)** EA GV26 + ION group occludin protein expression was significantly increased after treatment with MK-801, ***P < 0.001, GV26 + ION point group vs. GV26 + ION point + MK-801 group, *n* = 6/group. **(I)** The fluorescence continuity of occludin protein expression (green color, vascular morphology of brain endothelial cells) was more complete in the EA GV26 + ION group after the use of MK-801 (yellow arrows).

After clarifying the role of trigeminal nerve in promoting BBB opening, a preliminary investigation of the mechanism of BBB opening was performed based on EA of the GV26 + ION points. The expression of NR1 in the GV26 + ION point group was found to be significantly higher than that in the control group, with a statistically significant difference (*P* < 0.05), while the expression in the GV26 + GV20 group was slightly lower compared to the GV26 + ION point group, but the difference was not significant (*P* > 0.05) (Figure 4A). Changes in occludin protein expression indicate changes in the physical structure of the BBB, followed by changes in BBB permeability. We found that occludin expression was significantly lower in the GV26 + ION point group than in the control group, with a statistically significant difference (*P* < 0.05); the expression of occludin was slightly higher in the GV26 + GV20 group compared to the GV26 + ION point group, but without a significant difference (*P* > 0.05) (Figure 4B). We found that the EB content of the rat cortex was significantly increased in the GV26 + ION point group relative to the control group (*P* < 0.01) and the GV26 + GV20 group (*P* < 0.05), while the EB permeation through the cortical BBB was significantly reduced after the application of MK-801 in the NMDAR antagonist group, and the difference was statistically significant (*P* < 0.05) (Figures 4C,D). Figure 4E showed that EA GV26 + ION group cerebral cortex EB and c-Fos fluorescence expression was significantly decreased after use MK-801. We also found that NR1 protein expression was significantly lower in the GV26 + ION point + NMDAR antagonist group than in the GV26 + ION point group [the difference was statistically significant (*P* < 0.01)], and also lower than that in the GV26 + GV20 and control groups (but the difference was not statistically significant) (Figure 4F). Figure 4G shows the expression of NR1 was significantly decreased after use MK-801. We found that occludin protein expression was significantly higher in the GV26 + ION point + NMDAR antagonist group than in the GV26 + ION point group, and the difference was statistically significant (*P* < 0.001), but it was similar to the control group, which was not statistically significant (*P* > 0.05) (Figure 4H). Figure 4I shows the fluorescence continuity of vascular occludin was more pronounced in the GV26 + ION point + NMDAR antagonist group. This suggests that when the BBB is opened by EA stimulation of the ION point, the elevated expression of NMDA receptors may affect the expression of occludin, causing a decrease in occludin protein expression and poor vascular continuity, which in turn increased BBB permeability, the application of NMDA blocker MK-801 reverses this effect, allowing increased occludin protein expression and decreased BBB permeability.

### Effect of different intensities of electroacupuncture stimulation of the trigeminal nerve on blood-brain barrier opening and tight junction occludin

**FIGURE 5:**
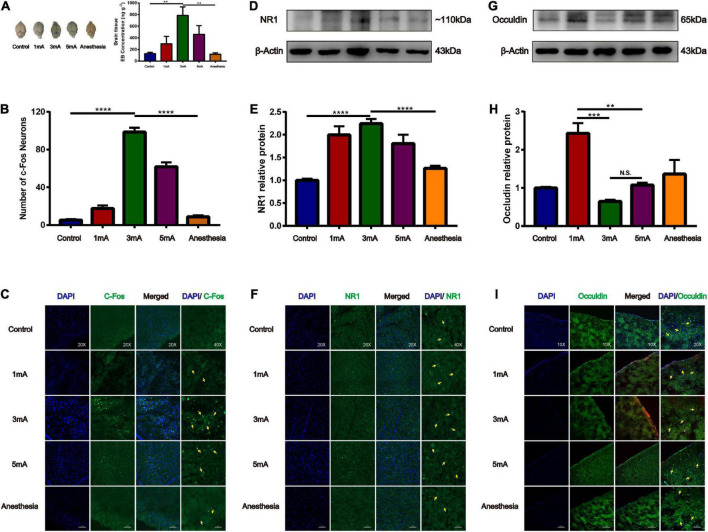
Different intensities of EA of trigeminal nerve on promoting BBB opening and its effect on tight junction protein occludin. **(A)** Shows the degree of brain EB penetration after different intensities of EA of trigeminal nerve under naked eye observation. EB concentration in the cerebral cortex; **P < 0.01, EA 3 mA group vs. control group and anesthesia (based on EA 3 mA) group; *n* = 6/group. **(B)** Number of c-Fos Neurons in the frontal cerebral cortex, four slices from each individual rat used for average; ****P < 0.0001, EA 3 mA group vs. control group and anesthesia group; *n* = 4/group. **(C)** Different intensities of EA of the trigeminal nerve, EA 3 mA group shows the most significant c-Fos fluorescence expression (yellow arrows). **(D,E)** Compared to the control group, NR1 protein expression was increased in all the EA groups, but the increase was most significant in the EA 3 mA group, and NR1 protein expression was decreased after anesthesia, ***P < 0.001, EA 3 mA group vs. control group and anesthesia group, *n* = 6/group. **(F)** Cerebral cortex NR1 fluorescence expression. The 3 mA group had the most pronounced expression. **(G,H)** The EA 3 mA group occludin protein expression was significantly decreased and no significant change was observed after anesthesia. But the EA 1 mA group had significantly increased expression, ***P < 0.001, 1 mA group vs. 3 mA group, **P < 0.01, 1 mA group vs. 5 mA group, *P* > 0.05, 3 mA group vs. 5 mA group, *n* = 6/group. **(I)** The EA 3 mA group occludin protein fluorescence expression was complete, with continuity after anesthesia (yellow arrows).

Our previous study found that at the same frequency of different current intensities (2, 3, 4, and 5 mA), EA stimulation of the GV26 + GV20 point had the greatest permeation effect at 3 mA and not at the maximum intensity of 5 mA, indicating that the degree of BBB opening are not linearly related. We found that the 3 and 5 mA groups had higher EB content than the 1 mA group, and the 3 mA group had higher EB content than the 5 mA group. Further, the 3 mA group had a significant difference in EB content compared to the control and anesthetized groups, which was statistically significant (*P* < 0.01) (Figure 5A). Figures 5B,C shows different intensities of EA trigeminal nerve, EA 3 mA group shows the most obvious c-Fos fluorescence expression compared to the control and anesthetized groups, which was statistically significant (*P* < 0.0001). We found compared with the control group, NR1 protein expression was increased in all EA groups, but the increase was most obvious in the EA 3 mA group compared to the control and anesthetized groups (*P* < 0.001), and NR1 protein expression was decreased after anesthesia (Figures 5D,E), Figure 5F shows NR1 fluorescence expression in the EA 3 and 5 mA groups was higher than that in the control, anesthesia, and EA 1 mA groups, this indicates that NMDA receptors change differently under different EA intensities, but not in a linear manner. We observed that occludin protein expression was significantly lower in the EA 3 mA and EA 5 mA groups than in the control, anesthesia, and EA 1 mA groups. The difference in occludin protein expression between the EA 3 mA and EA 1 mA groups was statistically significant (*P* < 0.001), and occludin protein expression was lower in the EA 3 mA group than in the EA 5 mA group, but it was not statistically significant (*P* > 0.05) (Figures 5G,H). Figure 5I shows that occludin expression continuity was better in the control, anesthesia, and EA 1 mA groups than in the EA 3 mA and EA 5 mA groups, 3mA group occludin protein fluorescence expression was still complete, continuity after anesthesia. The results showed that occludin protein morphology varied with different EA intensities.

### Effects of different intensities of electroacupuncture stimulation of the trigeminal nerve on changes in the excitability of neurons

**FIGURE 6:**
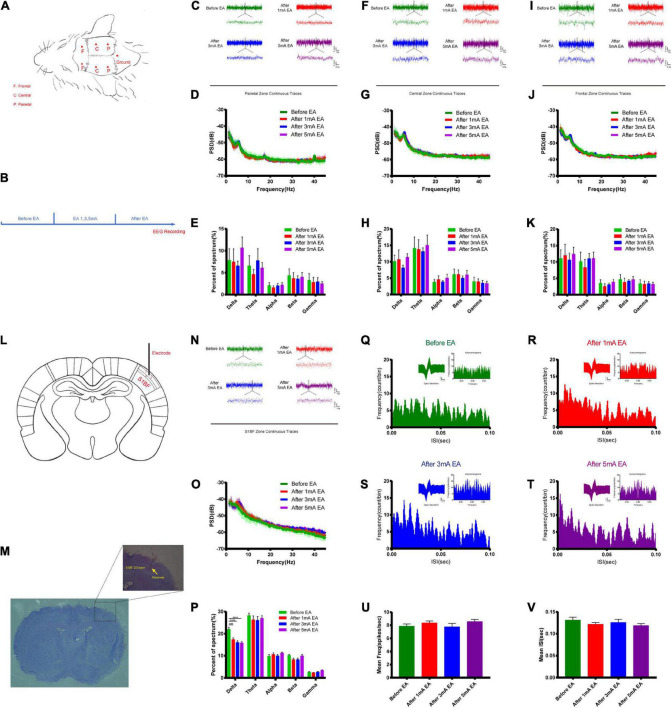
Effect of different intensities of EA of trigeminal nerve on neuronal excitability changes in EEG. **(A,B)** EEG detection location and experimental flow. **(C)** Parietal zone continuous traces, 100 s of EEG data were selected in the stable state without interference for before EA and after EA groups (the EEG data during EA could not be used because of too much interference from electromagnetic radiation) and 5 s of data were captured for display. **(D)** PSD (power spectral density) in the parietal zone before and after EA at different intensities. There was no significant difference between the groups. **(E)** Parietal zone of each group for delta 1–3 Hz, theta 3–7 Hz, alpha 7–12 Hz, beta 12–30 Hz, and gamma 30–45 Hz frequency band percent of the spectrum (%), which showed that there was no significant difference. **(F)** Central zone continuous traces, 100 s of EEG data were selected and 5 s of data were captured, for display. **(G)** The PSD central zone before and after EA of different intensities. There was no significant difference between the groups. **(H)** Central zone for each group frequency band percent of the spectrum (%); there was no significant difference for each group. **(I)** Frontal zone continuous traces, 100 s of EEG data were selected and 5 s of data were captured for display. **(J)** The PSD frontal zone before and after EA of different intensities. There was no significant difference between the groups. **(K)** Frontal zone group frequency band percent of the spectrum (%); there was no significant difference for each group. Zone frequency band percent of the spectrum (%) for each group showed that there was no significant difference for each group, *n* = 5/group. **(L,M)** Electrode implantation in S1BF 2/3 layer zone (corresponding to the infraorbital nerve stimulation point). **(N)** S1BF zone continuous traces, 100 s of EEG data were selected and 10 s of data were captured for display. **(O)** PSD before and after different intensities of EA in the S1BF zone. **(P)** The group frequency band percent of the spectrum (%) showed that delta rhythm decreased after EA of the S1BF zone, ***P < 0.001, control group vs. 1 mA group, ****P < 0.0001, control group vs. 3 and 5 mA groups. **(Q–T)** The inter spike interval (ISI, bin = 0.05 ms) for different intensities of EA stimulation in the S1BF zone showed a typical waveform, and autocorrelograms indicated that the frequency of short ISI (less than 50 ms) increased with the increase in EA intensity. **(U)** The mean frequency of spike in unit time second for different intensities of EA stimulation in the S1BF zone for each group is shown, which indicates that there was no significant difference between the groups. **(V)** The mean ISI time for each group after EA of the S1BF zone, which showed no significant difference between the groups, *n* = 4/group.

The opening of the BBB varies widely between EA and non-EA and between the intensity of EA and the level of neuronal activity represented by c-Fos; therefore, the electrical mechanisms behind different intensities of EA stimulation deserve further investigation to uncover possible electrical modulation patterns. Figures 6A,B shows a flowchart of the self-made cranial electrode model and the EEG experiment design. We found the continuous field potential trace maps and the power spectral density (PSD) before and after applying different EA intensities in the parietal region. There were no significant differences between the groups (Figures 6C–E). Figures 6F–H shows the central region continuous field potential trace maps, and the PSD indicated that there was no significant difference for each group. Figures 6I–K shows the frontal region continuous field potential trace maps and the PSD showed that there was no significant difference for each group before and after EA. This indicates that there was no significant change in all the frequency bands of LFP in the selected typical brain regions before and after EA. Figures 6L,M shows the schematic diagram of electrode implantation in the S1BF layer 2/3 area. We found that the delta rhythm decreased after EA in the S1BF area. There was a significant difference between the control group and the 1 mA group (*P* < 0.001), and a highly significant difference when the control group was compared to the 3 mA and the 5 mA groups (*P* < 0.0001), but there was no significant change in other frequency bands after EA (Figures 6N–P). Figures 6Q–T indicates that the frequency of short ISI (less than 50 ms) increased with the increase in EA intensity, but the typical waveform did not change. But we found that the average action potential (spike) frequency for different intensities of EA in the S1BF area was no significant difference between the groups (Figure 6U). Figure 6V shows the average ISI time of the entire recording cycle for each group in the S1BF area; again, no significant difference was found among the groups. This indicates that the delta rhythm of the low-frequency band of LFP decreased after EA, and the proportion of short ISI increased and changed more significantly with the intensity of EA. However, the number of spikes per unit of time and the mean ISI time varied little between the groups throughout the test.

### Effects of different intensities of electroacupuncture stimulation of the trigeminal nerve on the excitability of glutamatergic neurons in the S1 cortex

**FIGURE 7:**
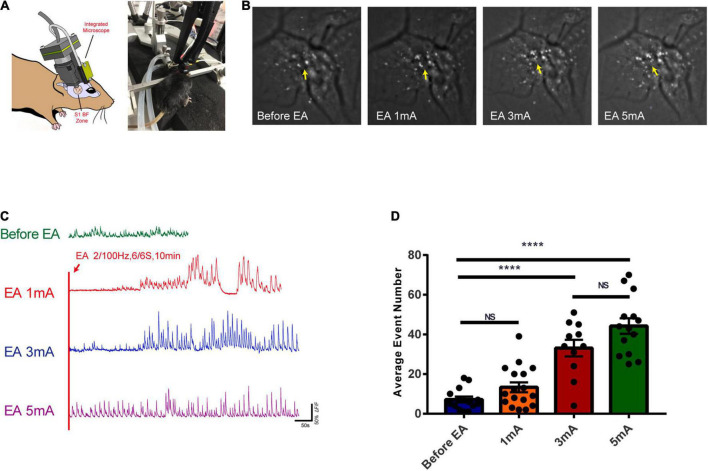
Effects of EA of the trigeminal nerve at different intensities on the excitability of glutamatergic neurons in cortex S1BF 2/3 layer. **(A)** *In vivo* calcium ion imaging pattern and experimental diagram. **(B)** Calcium accumulation map of glutamatergic neurons before and during EA at different intensities. **(C)** Traces map of glutamatergic neurons at different intensities. Traces map of glutamatergic neurons before and during EA at different intensities found that the calcium wave of glutamatergic neurons changed rhythmically with the intensity of EA. **(D)** The average calcium wave event number for each group found that the calcium wave event increased with the increase in EA intensity, ****P < 0.0001, before EA group vs. 3 mA and 5 mA groups; *P* > 0.05, before EA group vs. 1 mA group and 3 mA group vs. 5 mA group, *n* = 4/group.

The calcium ion activity of glutamatergic neurons was observed with different intensities of EA stimulation. Figure 7A shows the *in vivo* calcium ion imaging profile and the experimental diagram. The calcium accumulation map reflected the calcium ion accumulation increased significantly with increasing EA stimulation intensity, indicating a gradual increase in neuronal excitability (Figure 7B). The trace maps before EA and during different intensities of EA showed that glutamatergic neuronal calcium waves changed rhythmically with increasing EA intensity. There was no change before EA, while 1 mA, and 3 mA stimulation caused rhythmic changes after a period of EA stimulation, and 5 mA stimulation caused rhythmic changes at the beginning (Figure 7C and Supplementary Video 1). As shown in Figure 7D, the calcium wave events increased with the increase in EA intensity, and there was a highly significant difference in the before EA group compared to the 3 and 5 mA groups (*P* < 0.0001); further, there was no significant difference between the before EA group and the 1 mA group, and between the 3 and 5 mA groups (*P* > 0.05). This indicates that there is a critical point between 1 and 3 mA from the perspective of EA-induced neuronal excitability in the corresponding brain regions, after which neuronal excitability is significantly affected.

## Discussion

In a previous study, we found that after intravenous injection of EB, specific parameters of EA (2/100 Hz, 3 mA, 6/6 s, 40 min) at the head GV26 and GV20 acupuncture points could promote the permeation of EB through the BBB and uptake by the neurons (Zhang J. et al., 2018; Zhao et al., 2019). However, EA of non-head acupuncture point, ST36, had no cortical EB permeation effect in rats, indicating that the location of selected acupuncture points is also important. According to modern physiological and neuroanatomical views, both the GV26 and GV20 points are located within the sensory distribution area of the trigeminal nerve; therefore, the research team further investigated whether EA of the GV26 and GV20 points opens the BBB through the trigeminal nerve.

The trigeminal nerve is one of the thickest mixed cranial nerves in the human body, and a close relationship exists between the trigeminal nerve and cerebral vasculature. Modern studies have found that the trigeminal nerve affects the function of the cerebral vasculature mainly through the following pathways (Chiluwal et al., 2017; Yu et al., 2017). The first pathway is through the trigeminal ganglion after stimulation of the meningeal branch fibers; the nerve endings wrapped around the dural vessels secrete substance P and calcitonin gene-related peptide (CGRP) among others, to influence the permeability of the meningeal vessels. The second pathway is through the pterygopalatine ganglion, through the trigeminal-parasympathetic reflex arc, affecting the cerebral vasculature (Petersen, 2007; Just et al., 2010). Wang et al. (2020) believe that head acupuncture points are mainly innervated by the trigeminal nerve and the subcranial meningeal vessels through axonal branches of primary neurons of the trigeminal ganglion, through gradual synaptic projections from the periphery to the center, through secondary neurons of the trigeminal spinal nucleus to the thalamus and cortex, and through postsynaptic neurogenic convergence and integration, to exert various physiopathological regulatory functions.

Therefore, we selected the non-trigeminal sensory distribution area of the double-clamp ear (EX-HN6) acupuncture point and the optimal stimulation point of the largest branch of the trigeminal nerve, the infraorbital nerve (located in the right cheek of rats, the first 2/3 of the lower edge of the zygomatic bone), and performed EA stimulation with specific parameters. We hypothesized that EA stimulation of head acupuncture points to promote BBB opening might be mainly conducted from the trigeminal nerve to the central cortex. Second, to verify whether the infraorbital nerve promoted BBB opening after EA, the group performed excisional modeling of the infraorbital nerve and found no EB permeation effect after EA. This indicates that the conduction of EA stimulation from the infraorbital nerve was interrupted, and thus, there was no BBB opening effect. Since the trigeminal nerve has multiple pathways to the cerebral vasculature, a trigeminal ganglion block was performed to avoid interference from the meningeal branch and pterygopalatine ganglion, followed by EA stimulation of the GV26 + infraorbital nerve stimulation point. This suggests that EA stimulation is conducted from the peripheral infraorbital nerve to the trigeminal ganglion and possibly to the center via synaptic projections, mediating excitatory changes in neurons.

Sonnay et al. (2017) also used infraorbital electrical nerve stimulation to monitor changes in blood oxygen and neurotransmitter metabolism in the corresponding brain regions in real time using functional Magnetic Resonance Imaging (fMRI) and found that blood oxygen level-dependent (BOLD) effects were observed in the primary somatosensory cortex (S1BF), the secondary somatosensory cortex (S2), and the motor cortex (M1) to which stimuli were delivered via synapses (Just et al., 2010, 2013). The BOLD effect was observed in S2 and in M1, where these effects were obtained through the synaptic projection of the stimulus. Therefore, we suggest that the EA GV26 + infraorbital nerve stimulation point, which directly stimulates the largest branch of the trigeminal nerve, projected gradually through the trigeminal axons to the central cortex and other corresponding brain regions, thereby inducing neuronal activity based on the secretion of neurotransmitters, resulting in a BBB opening effect, probably through the effect of neuronal NMDA receptors on the cerebral cortex (Lu et al., 2019). The BBB opening effect may be produced by binding glutamate transmitters released after EA stimulation, which in turn affect tight junction proteins on brain endothelial cells through a series of signaling pathways (Chen et al., 2016) in the brain endothelium, especially the occludin protein (Furuse et al., 1993; Dorfel and Huber, 2012). The physical structure of the BBB is altered, which in turn affects the degree of BBB opening.

*N*-methyl-D-aspartate receptors are one of two major classes of postsynaptic glutamatergic ionotropic channel receptors that mediate the slow component of excitatory synaptic permeation. They are dual voltage-gated and chemically gated channels, with NR1 as an essential subunit. Occludin, in the CNS, is morphologically a four-span transmembrane protein expressed in vascular cells; it is the first transmembrane protein specifically localized to the TJ and anchors with ZO-1 (Fanning et al., 1998) and ZO-2. Occludin plays an important role in stabilizing the physical structure of the BBB similar to the opening and closing of the gate valve (Furuse et al., 1993; Buschmann et al., 2013). Many research teams have also found that glutamate release activates NMDA receptors in the cerebral cortex; through a complex series of signal transduction pathways (Sun et al., 2005; Hannah et al., 2011), glutamate release increases the inward flow of calcium ions to brain endothelial cells, reduces brain endothelial cell transendothelial resistance and occludin expression levels, alters occludin structure, and results in increased microvascular permeability in the rat cerebral cortex (Dorfel and Huber, 2012; Chen et al., 2016). In this study, we also found that NR1 expression was significantly increased in the EA GV26 + infraorbital nerve stimulation point group compared to other groups, including the GV26 + GV20 group, indicating that there was a trend of differential expression of NR1 in each group. To some extent, this suggests that EA stimulation of infraorbital nerve stimulation point can induce an increase in NMDA receptor expression, which is associated with increased neuronal excitability, increased secretion of glutamate transmitters, and NMDAR receptor binding. As for occludin protein expression in each group, it was found that occludin expression was significantly lower in the EA GV26 + infraorbital nerve stimulation point group than in the other groups, which also indicated a trend of differential expression of occludin in each group. In contrast, when EA was performed after application of the NMDAR receptor antagonist MK-801 (Neuhaus et al., 2012) in the group with the GV26 + infraorbital nerve stimulation point, the permeation of EB content through the cortex BBB was significantly lower, NR1 protein expression was significantly reduced, occludin protein expression was significantly restored, and the immunofluorescence showed intact vascular continuity. During the signal transduction process involving NMDA receptors, the downstream expression of the tight junction protein occludin and the physical structure of the BBB were altered, which in turn affected its permeability, while MK-801 reversed the action of NMDA receptors. It also indirectly indicates the role of central NMDA receptors in BBB opening mediated by infraorbital nerve EA stimulation (Chen et al., 2016).

A previous study found that at the same frequency of different current intensities (2, 3, 4, and 5 mA) of GV26 + GV20 acupoint stimulation, 3 mA and not the maximum 5 mA current intensity had the greatest effect on BBB opening, suggesting that neuronal activity and BBB opening are not linearly dependent and that there may be an underlying mechanism that allows neuron activity induced by EA acupoint stimulation to be within the threshold of the BBB opening effect (Chen et al., 2019). An earlier study found that while using ultrasound stimulation to activate neurons (Baseri et al., 2012; Cui et al., 2019) and open the BBB, different intensities of ultrasound stimulation resulted in different degrees of BBB opening. Low-intensity ultrasound (0.12 MPa) stimulation did not lead to BBB opening, but resulted in the activation of c-Fos, whereas when the intensity was increased to 0.25 MPa, there was EB permeation through the BBB, indicating that the neurons are activated to produce excitatory changes that must reach a certain level to induce changes in BBB permeability (Chen et al., 2019). The experiment was performed with anesthesia (Fu et al., 2017) and the intensity of EA was gradually increased; the EB permeation effect was better in the 3 and 5 mA groups than in the 1 mA group, and moreover, the permeation effect was better in the 3 mA group than in the 5 mA group, indicating that the BBB opening promoted by the different intensities of EA stimulation is not a linearly dependent relationship. The NR1 protein expression and fluorescence intensity were highest in the 3 mA group and the occludin protein expression and fluorescence vascular continuity were the lowest; however, the occludin protein expression in the 1 mA group was significantly elevated for reasons that need further elucidation and is possibly related to the bidirectional effect of EA.

Because of the differences in BBB opening between the EA and non-EA groups, as well as the differences in the intensity of EA and the degree of neuronal activity represented by c-Fos, the electrical mechanisms behind the different intensities of EA stimulation deserve further investigation. There may be certain electrical modulation patterns, and our study revealed new electrical phenomena. The EEG of different typical brain regions (parietal, central, and frontal regions) during EA was first measured with self-made electrodes, and the EEG before and after EA was recorded immediately (the EEG during EA was not available due to excessive electromagnetic radiation; therefore, the *in vivo* calcium ion imaging technique was applied afterward to supplement the observation during EA). The PSD in each frequency band (delta 1–3 Hz, theta 3–7 Hz, alpha 7–12 Hz, beta 12–30 Hz, and gamma 30–45 Hz) were not significantly different, which is consistent with our previous study, which found that EA opening of the BBB is transient and reversible at the electrophysiological level. After EA, neuronal activity was significantly reduced, and there were no significant ongoing after-effects. However, our subsequent modeling of the implanted electrode, with EEG recordings from S1BF brain regions corresponding to the infraorbital nerve stimulation points, revealed subtle changes in individual neuronal electrical activity at the microscopic level. First, it was found that the LFP delta rhythm decreased after EA compared to before EA, and the difference was more significant with increasing EA intensity. However, there was no significant change in other frequency bands after EA, suggesting that EA stimulation may transiently reduce the discharge level in the lower frequency bands but does not have a sustained effect on the higher frequency bands. Second, the S1BF area with different intensities of EA ISI, as well as typical waveforms and autocorrelation plots, found that the frequency of short ISI (less than 50 ms) increased with the increase in EA intensity, but the typical waveforms did not change. There was no significant difference in the average spike frequency of each group with different EA intensities, as well as the average ISI time of the entire recording cycle for each group. This indicates that the proportion of action potential (spike) short ISI increases and changes more significantly with EA intensity. However, for the entire cycle, the number of spikes per unit time and the average ISI time did not change significantly among the groups, and the correlation and deeper data analysis remain to be elucidated. *In vivo* calcium imaging was applied to observe changes in calcium flow in glutamatergic neurons in layer 2/3 of the S1BF area during EA, and it was found that glutamatergic neuronal calcium wave energy changed rhythmically with increasing EA intensity. There was essentially no change before EA; 1 and 3 mA caused rhythmic changes after a period of EA stimulation, while 5 mA stimulation showed rhythmic changes at the beginning. This is different from the previous observation of the BBB opening effect, which found that 3 mA had the most significant effect on BBB opening for reasons that remain unknown; but, it is consistent with the fact that the higher the EA intensity, the faster the calcium wave rhythmicity appears. In addition, from the perspective of EA-induced neuronal excitability in the corresponding brain regions, there is a threshold between 1 and 3 mA, after which neuronal excitability and BBB permeation are significantly affected.

In summary, this study investigated the role of the trigeminal nerve in EA stimulation of different acupuncture points in the head, based on the effect of EA stimulation on BBB opening, and made a preliminary exploration of the possible mechanisms. We found that NMDA receptors play a mediating role in the trigeminal nerve conduction of peripheral EA stimulation to the center, and affect the expression of the tight junction protein occludin, leading to BBB opening. We also explored the possible electrical mechanisms underlying EA stimulation based on different intensities of EA of the trigeminal nerve and found that EEG immediately before and after EA did not show significant changes in the major brain areas, but there was a decrease in the low-frequency band of LFP and an increase in the percentage of short ISI in the local trigeminal nerve corresponding to S1BF. However, the number of spikes per unit time and average ISI time did not change significantly across the EEG recording cycle. We applied the *in vivo* calcium ion technology to observe the changes in the excitability of glutamatergic neurons during EA and found that the rhythmic changes of calcium waves became more pronounced with increasing intensity of EA. However, this rhythmic alteration produced calcium ion fluctuations, although it reflected the altered excitability of underlying neural activity. The intrinsic association with BBB opening needs to be further explored (Sun et al., 2013), and the role of calcium fluctuations in BBB opening during EA in various types of interneurons and astroglial cells deserves further elucidation. The results indicate that BBB opening by trigeminal EA stimulation was targeted at the level of detection of altered excitability of neural activity following EA stimulation of the GV26 and GV20 points, and found that CNS cortical c-Fos, NMDA receptors (Sharp et al., 2005; Reijerkerk et al., 2010), and occludin were altered; however, the integrity of the relationship between upstream and downstream signaling and BBB opening is worth exploring. It is also possible to identify the relevant signaling pathways through genetic and proteomic analysis (Ma et al., 2022), such as the Wnt and the astrocyte Shh pathway, which will open up new ideas and validate specific mechanisms of action of EA at the molecular and cellular levels in combination with chemical, genetic, and optogenetic methods.

## Conclusion

The trigeminal nerve plays an important role in EA stimulation of head acupuncture points to promote alterations in BBB permeability. Brain NMDA receptors play a mediating role during EA of the trigeminal nerve to promote BBB opening, affecting the expression of the tight junction protein occludin, which in turn alters BBB permeability. BBB opening was not linearly dependent on EA intensity. Immediately after EA of the trigeminal nerve at different intensities, no significant changes were detected in each rhythmic band of field potentials in different brain regions, which is consistent with the conclusion that BBB opening by EA is transient and reversible at the electrical level. However, EA of the local S1BF area corresponding to the trigeminal nerve resulted in a transient decrease in the delta rhythm of LFP and no changes in the high-frequency band. The short ISI of action potentials varied with the intensity of EA, but the mean frequency of spikes per unit time and the mean ISI did not change significantly. Specific parameters of EA of the trigeminal nerve resulted in rhythmic changes in the calcium flow of glutamatergic neurons in the S1BF region, which varied with the intensity of EA stimulation. This provides a research perspective and technical approach to further explore the mechanism of EA in BBB opening and its potential clinical application.

## Data availability statement

The original contributions presented in this study are included in the article/Supplementary material, further inquiries can be directed to the corresponding author/s.

## Ethics statement

The animal study was reviewed and approved by Animal Ethical and Welfare Committee of ZCMU. Written informed consent was obtained from the owners for the participation of their animals in this study.

## Author contributions

PG, JZ, and XL conceived and designed the experiments. PG, SZ, LR, YZ, XM, HW, LG, CM, YL, QY, and KQ performed the experiments. PG, LR, and LG analyzed the data. PG and SZ drafted the manuscript. XL and JZ revised the manuscript. All authors contributed to the article and approved the submitted version.

## Supplementary material

Supplementary Video 1 *In vivo* calcium imaging recording video. This video showed glutamatergic neurons in cortex S1BF 2/3 layer before and during EA at different intensities, found that the calcium wave of glutamatergic neurons changed rhythmically with the intensity of EA.
